# Rare case report: a case of histological type transformation of lung cancer caused by neoadjuvant immunotherapy

**DOI:** 10.3389/fonc.2024.1329152

**Published:** 2024-02-15

**Authors:** Quanqing Li, Guangxin Zhang, Hao Yang, Jindong Li

**Affiliations:** Department of Thoracic of the Second Hospital of Jilin University, Changchun, Jilin, China

**Keywords:** histological type transformation, neoadjuvant immunotherapy, small cell lung cancer, drug resistance, squamous cell lung carcinoma

## Abstract

Lung cancer remains the leading cause of cancer-related mortality, with 1.8 million deaths per year. Small cell lung cancer and non-small cell lung cancer (NSCLC) are the main cancer types. Approximately 85% of cases are NSCLC, including adenocarcinoma, squamous cell carcinoma, and large cell carcinoma. In this reported treatment case, the tumor histological type changed after targeted therapy, which has not been previously well documented. The patient was a 67-year-old woman diagnosed with squamous cell carcinoma via bronchoscopy. She received five neoadjuvant immune monotherapies. The lesion shrank but then progressed, with a diagnosis of small cell carcinoma via bronchoscopy. This finding suggests that tumor acquisition of resistance as manifested by cancer-type changes needs consideration and study in the application of this particular type of immunotherapy.

## Introduction

Immunotherapeutic agents such as sintilimab (anti-PD-1) are antibodies that promote immune system activation and exhibit good efficacy in the first-line treatment of non-small cell lung cancer (NSCLC). Squamous cell carcinoma responds very well to this preoperative immunotherapy (1). Some reactions such as fever and fatigue are observed, most of which are primary or secondary treatment-related adverse events (TRAEs) (2), although serious grade III TRAEs are rare. A related study has revealed that histological transformation into small cell lung cancer (SCLC) from NSCLC is a potential mechanism in therapeutic resistance (3). Herein, we describe the relevant medical history, examination and diagnosis, and treatment regimens of a patient to improve our understanding of this disease, avoid potential misdiagnosis, and provide a basis for a more standardized care of lung cancer patients.

## Case presentation

**Figure 1:**
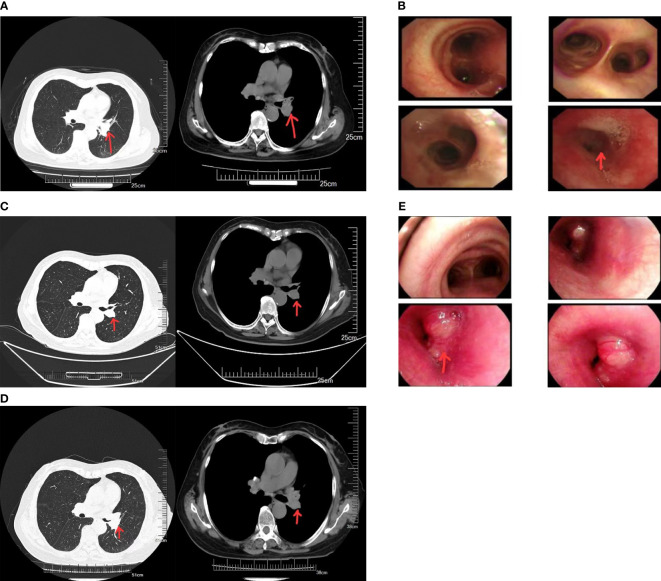
**(A)** Computed tomography (CT) images of the patient before neoadjuvant therapy. **(B)** Results of the first bronchoscopy. **(C)** CT images of the patient after three cycles of sintilimab immunotherapy. **(D)** CT images of the patient after five cycles of sintilimab immunotherapy. **(E)** Results of the second bronchoscopy.

The patient was a 67-year-old woman. Seven months ago, she exhibited no obvious reasons for cough or white sputum. At times, she coughed up blood. She did not receive systematic diagnosis and treatment. One month later, she felt suffocation in the anterior chest area with poor breathing; again, she did not pay attention to these signs. Only after the gradual worsening of the symptoms did she visit our hospital. Chest computed tomography (CT, **Figure 1A**) revealed the presence of a soft tissue mass in the left hilum of her lung. The lesion was 32 × 25 mm in size, with uneven density and a CT value of 32 HU. It was protruding into the bronchus of the upper lobe of the left lung. There was bronchial wall thickening, distal lumen obstruction, and many mediastinal lymph nodes. Bronchoscopy (**Figure 1B**) revealed mucosal swelling at the opening of the left upper lobe, superficial irregular hyperplasia, lumen occlusion, lesions involving the upper and lower interlobar ridges, and lumen stenosis at the opening of the left lower lobe. Pathological examination of the opening of the left upper lobe revealed a tumor morphology consistent with squamous cell carcinoma (non-keratinizing type). The patient had a smoking history of 20 years and a smoking index of 400 years. A physical examination found no obvious abnormality. Tumor marker (sample number 20230324HYA001) analysis showed the following: abnormal prothrombin, 39.740 mAU/ml (reference value: 11.12–32.01 mAU/ml); cytokeratin 19 fragment at 2.42 ng/ml (reference value: 0–2.08 ng/ml); and premenopausal ROMA value of 12.00% (reference value: 0%–7.4%); the other markers were normal. Head-enhanced magnetic resonance imaging, abdominal CT, whole-body bone scan, and other auxiliary examinations suggested no metastasis or surgical contraindications. The tumor stage was T2aNxM0, and the clinical stage was IB as per the guidelines for primary lung cancer diagnosis and treatment of the China Health Commission (2022 edition). As a result, surgery was indicated. The preoperative pulmonary function test suggested mild obstructive ventilatory dysfunction. However, after being informed of the surgical risks, the patient and her family declined surgery. As a substitute, preoperative chemotherapy combined with neoadjuvant immunotherapy sintilimab and gemcitabine–platinum-containing drug for squamous cell carcinoma was prescribed as per the first-line drug treatment guidelines for primary lung cancer diagnosis and treatment (2022 edition). Prior to chemotherapy, the patient scored 50 on the Karnofsky Performance Scale (KPS), which precluded chemotherapy, and only immunotherapy was administered. Due to economic issues, the PD-1 test was not performed. In the end, sintilimab immunotherapy was carried out for 21 days as one cycle. The lung cancer guidelines do not specify the number of immunotherapy cycles. The patient’s symptoms significantly improved after three cycles. Chest CT (**Figure 1C**) showed that the tumor size was reduced to 21 × 24 mm. From the good result of neoadjuvant immunotherapy, surgery was recommended again. Yet, the patient and her family refused it again due in part to the good effects of immunotherapy, plus cost consideration and other related reasons. Therefore, maintenance treatment was administered as per the primary lung cancer diagnosis and treatment guidelines, which can be selected for patients who attain disease control after first-line treatment. If no disease progression and tolerable adverse reactions are obtained from using these immune checkpoint inhibitors, these treatment cycles can be administered for 2 years. However, after the fourth cycle of sintilimab, the symptoms began to worsen. Before the fifth cycle, chest CT (**Figure 1D**) revealed that the tumor size had increased to 31 × 24 mm. Bronchoscopy (**Figure 1E**) revealed that the lumen was blocked by new growth in the lower portion of the left main bronchus, with an irregular layer of cell proliferation. The lower portion of the left main bronchus was biopsied. Pathology revealed bronchial carcinoma. Immunohistochemical staining combined with morphology supported the diagnosis of small cell carcinoma. At this time, the patient scored 60 on KPS. Considering that the patient could likely tolerate the side effects of chemotherapy, etoposide plus nedaplatin was recommended. After chemotherapy, chest high-resolution CT revealed a reduced lesion size of 20 × 18 mm, suggesting that the tumor had responded to the new treatment regimen.

## Discussion

Currently, drug resistance arising during treatment is a major treatment-related concern for patients with NSCLC. We summarized some differences in treatment resistance between the use of epidermal growth factor receptor tyrosine kinase inhibitors (EGFR-TKIs) and anti-PD-1, as well as the mechanisms in tumor histology changes as a result to provide insight for counteracting drug resistance in NSCLC treatment in the future.

### Transformation of NSCLC into SCLC and T790M point mutation in EGFR-TK during EGFR-TKI treatment

In patients with active EGFR, EGFR-TKIs can rapidly shrink the primary tumor; nevertheless, resistance appears in approximately 12 months (4, 5). The most obvious reason for resistance is the T790M point mutation in exon 20 that increases ATP affinity (6). The other is the transformation of EGFR-positive NSCLC into SCLC (7, 8). In a retrospective study, 58 patients with NSCLC and EGFR mutations were enrolled. Among them, 93% of the patients received EGFR-TKIs; all patients received a median of more than two lines of treatment. Ninety-seven percent of the patients were found to harbor SCLC (9). Significantly, the original activated EGFR mutation was retained in the tumor tissues that transformed into SCLC, suggesting a direct lineage from NSCLC rather than sampling or an undiagnosed primary lesion (10). RB1 inactivation is an important marker for SCLC. Western blotting of repeated biopsy samples from patients with EGFR mutation-positive adenocarcinoma transformed into SCLC showed the absence of RB1 in all cases and not in those with EGFR mutation-positive NSCLC (11). However, RB1 expression loss or downregulation alone via experimental manipulation on TKI-resistant cancer cell lines with EGFR mutations did not result in NSCLC transformation into SCLC. EGFR-TKI treatment plus genetic mutations, such as RB1 and TP53 deletions, may act in concert to promote tumor differentiation into SCLC (11, 12).

### Transformation of NSCLC into SCLC during PD-1 treatment

Anti-PD-1/PD-L1 interaction has become the paradigm of immune checkpoint inhibitor-based cancer treatment. It has successfully prolonged the survival of patients with advanced NSCLC. When tumor cells are detected by the body’s immune system, proinflammatory molecules, chemokines, and innate immunity cells are congregated at the tumor sites causing antitumor responses (13). PD-1 is expressed in T cells, whereas PD-L1 is expressed in tumor cells. The PD-1/PD-L1 axis inhibits T-cell function (14), a process that could be interrupted by PD-1 antibodies (14, 15). A study has revealed that the emergence of PD-1 treatment resistance might be due to defects in antigen processing and presentation by tumor cells with the result that the immune system is no longer able to detect the tumor antigens and initiates tumor cytolysis. Human leukocyte antigen class I (HLA-I) can bind to specific peptides of intracellular proteins, express them on the cell surface, and present them to CD8^+^ T cells. β2-microglobulin (B2M) is needed to maintain stable HLA-I expression, and mutations in B2M can hamper antigen presentation. Therefore, impairment in HLA-I-mediated antigen presentation could lead to resistance to checkpoint inhibitors (16). Therefore, the transformation of NSCLC into SCLC with defects in antigen presentation may be another route to resistance.

The treatment-induced transformation of adenocarcinoma into small cell carcinoma merits further investigation (17). Understanding the possible origin of the different lung cancer types is essential. In general, SCLC cells express neuroendocrine markers, such as neuron-specific enolase and progastrin-releasing peptide. Tumor cells with neuroendocrine expression could be derived from airway neuroendocrine cells localized in the central area near the lung hilum (18). Adenocarcinoma cells are derived primarily from type II pneumocytes, and carcinoma cells with squamous differentiation from basal cells (19). Other studies have reported that EGFR mutation-positive adenocarcinoma cells can also be derived from alveolar type II cells. In fact, alveolar type II cells can produce both adenocarcinoma and SCLC (19–21). Therefore, EGFR mutation-positive lung cancer occurring in alveolar type II cells may be readily transformed into SCLC (7).

In the present case report, the patient is a woman with a long smoking history. Smokers are particularly susceptible to squamous cell carcinoma and small cell carcinoma (22, 23). The treatment regimens are different for these two cancer types. As per the primary lung cancer diagnosis and treatment guidelines (2022 edition), surgery remains the primary treatment modality for resectable squamous cell lung carcinoma. However, our patient forwent surgery for the reasons given above. In patients with advanced or unresectable NSCLC, patients undergoing PD-1 treatment showed longer progression-free survival, overall survival, and fewer adverse events than those undergoing platinum-based chemotherapy (24). Therefore, we chose the PD-1 inhibitor monotherapy to treat that patient. Encouraging results were obtained in the first three cycles of therapy, but the patient developed worsening symptoms and tumor growth after four cycles. We suspected tumor resistance or tumor hyperprogression needing clarification by pathology. Testing revealed small cell carcinoma suggesting a change in the histological type induced by therapy. We searched the relevant literature and retrieved 10 reports, where eight patients received nivolumab and two received pembrolizumab; these patients also received chemotherapy before immunotherapy (25). All these patients exhibited tumor histological type transformation after treatment. For patients with treatment-induced SCLC, no clear guidelines are available on whether further application of immunotherapeutic drugs will benefit them. Etoposide combined with platinum remains the primary treatment modality for SCLC (26). For this patient, we chose this method as the next treatment option. The prompt discovery of SCLC transformation prevented unnecessary surgical trauma and wrong medication which might aggravate her well-being. The pathogenesis of transformed SCLC should be explored. To date, no explanation is available on the specific mechanism underlying this phenomenon.

In the present case, another reason for the histological results of the two samplings might be the uncertainty of missing SCLC diagnosis in the preliminary pathology. Previous studies have found that 9 (2%) of 429 patients with SCLC had a combined subtype of small cell carcinoma plus squamous cell carcinoma or adenocarcinoma at the time of diagnosis (27). Existing clinical data and evidence suggest that SCLC has a shorter survival time with fewer choices of effective therapeutic intervention than NSCLC. Transformed SCLC is a result of tumor resistance (28). Extensive clinical research suggests that it is not rare for the transformation of NSCLC to SCLC when therapeutic drugs are used.

## Conclusion

We present the case of a woman with dyspnea symptoms from central lung cancer. The first bronchoscopy revealed squamous cell carcinoma. After five cycles of sintilimab, re-examination revealed small cell carcinoma. Whether surgical resection, if carried out at first, could affect this patient’s disease course is unknown.

## Data availability statement

The original contributions presented in the study are included in the article/supplementary material. Further inquiries can be directed to the corresponding author.

## Ethics statement

The studies involving humans were approved by the Ethics Committee of the Second Hospital of Jilin University. The studies were conducted in accordance with the local legislation and institutional requirements. The participants provided their written informed consent to participate in this study. Written informed consent was obtained from the individual(s) for the publication of any potentially identifiable images or data included in this article.

## Author contributions

QL: Writing – original draft, Conceptualization. GZ: Supervision, Writing – review & editing, Validation. HY: Investigation, Writing – original draft. JL: Writing – review & editing, Validation.
